# Intention to use institutional delivery service and its predictors among pregnant women, North West Ethiopia: Using theory of planned behavior

**DOI:** 10.1371/journal.pone.0248697

**Published:** 2021-05-07

**Authors:** Abirham Ayana, Ayenew Kassie, Telake Azale

**Affiliations:** 1 Amhara Regional State Health Bureau, Bahir Dar, Ethiopia; 2 Department of Health Education and Behavioral Sciences, College of Medicine and Health Science, University of Gondar, Gondar, Ethiopia; University of Mississippi Medical Center, UNITED STATES

## Abstract

**Background:**

Improving institutional delivery service is the most crucial strategies to reduce maternal and neonatal mortalities. In developing countries, only 50% of pregnant women deliver in health facilities and in Ethiopia only 48% of pregnant women deliver in health facilities. Maternal mortality remains the highest in Ethiopia. This study assessed intention to use institutional delivery service and its predictors among pregnant women using theory of planned behavior.

**Methods:**

Community-based cross-sectional study was conducted among 645 Yilmana Densa District Pregnant women using multi-stage followed by cluster sampling technique. Data were entered into Epi Data version 4.6.0.2 and analyzed with STATA version 14. Binary logistic regression analysis was done to identify independent predictors of intention at 95% confidence level and P < 0.05 was used to determine statistically significant predictors.

**Results:**

Intention of pregnant women to use institutional delivery service was 74.3% (CI; 70.71%, 77.6%). In the multivariable logistic regression; those who had 1–3 and 4 &above antenatal care 2.85(1.41, 5.75) and 3.14(1.16, 8.45) respectively, those who had past experience of institutional delivery (AOR = 3.39, 95%CI: 1.72, 6.71), parity of 1–3 and 4 & above % (AOR = 0.37, 0.19, 0.71) and (AOR = 0.25, 95%CI: 0.12, 0.55) respectively, rural residence (AOR = 0.51, 95%CI: 0.27, 0.96), favorable attitude (AOR = 2.93, 95%CI: 1.56, 5.50), favorable perceived behavioral control (AOR = 2.60, 95%CI: 1.44, 4.69) were factors significantly associated with intention to use institutional delivery service.

**Conclusion and recommendation:**

Majority of the pregnant women were intended to deliver in the institution. Good Knowledge on institutional delivery, antenatal care visit, past experience of institutional delivery, rural residence, parity, attitude and perceived behavioral control were identified factors significantly associated with intention to use institutional delivery service. So, strengthening awareness creation and behavioral change communication programs are required at all levels of health system to raise intention of residents towards institutional delivery.

## Introduction

Institutional delivery is childbirth taken place in health facilities conducted by skilled birth attendants [1]. The World Health Organization (WHO) reported that only 50% of pregnant women in developing countries delivered by skilled birth attendants [2]. Even if maternal mortality reduces from time to time, around 800 pregnant women die due to pregnancy and delivery-related complications daily worldwide. Nearly 99% of maternal and neonatal mortality occurs in low and middle-income countries [2, 3].

In sub-Saharan Africa (SSA), the maternal mortality ratio in 2013 is 510 deaths per 100,000 live births [4] and the leading causes of maternal death are; Antepartum Hemorrhage (APH), Postpartum Hemorrhage(PPH), pregnancy-induced hypertension (eclampsia, preeclampsia), unsafe abortion, obstructed labor and sepsis [5, 6]. In Ethiopia, maternal mortality remains among the highest in the world. According to the Ethiopian demographic health survey (EDHS) 2016 report, maternal mortality is 412 per 100,000 live births [6, 7]. Ethiopia is one of the sub–Saharan African countries which has low institutional delivery care utilization among pregnant mothers [8]. Institutional delivery is one of the most important strategies to reduce maternal and neonatal mortalities. However, the coverage of health facility delivery is low. In developing countries, many pregnant women are not assisted by skilled birth attendants even if they have at least one ANC follow up. A recent report from the Tanzania Ministry of Health and social welfare indicated that more than 90% of pregnant women have at least one antenatal care visit, but only 62% of pregnant women delivered at a health facility in 2012 [9, 10].

In Ethiopia, 74% of pregnant women have received antenatal care from a skilled provider at least once, from a doctor, nurse, or midwife, for their current pregnancy. However, institutional delivery is the lowest in the world. According to EDHS 2016 and Mini EDHS 2019 and health facility delivery is 26% and 48% respectively, which is unacceptably low [7–11]. Studies conducted in different studies in showed that the magnitude of intention to utilize institutional delivery ranged from 30.3% to 75.5% [12–14].

The theory of planned behavior (TPB) is a psychological theory that predicts an individual’s intention to engage in a behavior at a specific time and place. The central factor in the TPB is the individual’s intention to perform a given behavior. It has three direct determinants of intention these are attitude, subjective norm, and perceived behavioral control [15]. It has also an indirect predictor of intention these are behavioral belief and outcome evaluation, normative, and motivation to comply and control belief and power [15, 16].

Literatures in Ethiopia showed the magnitudes of the pregnant mother’s intention to deliver in health facilities. For example, in Debre markos 74.3% and Wolaita soda 75.5% pregnant women planned to deliver at health institutions [18, 19].

Studies conducted in Nepal and different part of Ethiopia showed that younger pregnant women were more intended to utilize institutional delivery [12, 14, 17–20]. In different studies it is revealed that maternal education determine intention to utilize institutional delivery [17, 21]. The residence, occupation, income, religion could affect pregnant mothers intention [13, 17, 22].

Studies done in Tanzania, Wollaita Soddo town and Awash Fetale district revealed that pregnant women who had positive attitude towards institutional delivery were more intended to use institutional delivery than women with negative attitude [13, 23, 24]. Studies conducted in Zambia and Gedeo zone showed that favorable subjective norm and favorable perceived behavioral control had positive association to intention use institutional delivery [13, 21, 25]. However, other some studies showed that there is no intention difference between behavior beliefs [26]. Therefore, this study assessed intention to use institutional delivery and its predictors among pregnant mothers in Yilmana Densa district using theory of planned behavior.

## Methods

### Study design and settings

Community-based cross-sectional study design was conducted from 15^th^ February 2020 to 14^th^March 2020. It was conducted in Yilmana Densa district, which is one of the districts found in west Gojam zone, Ethiopia. It is found 485 km away from Addis Ababa, which is the capital city of Ethiopia [27]. The total number of pregnant women identified in the selected clusters during the data collection period was 805.

### Study population and sampling

All permanent resident pregnant women were included from the selected clusters of Yilmana Densa district. However, those who were severely ill who couldn’t give information were excluded. The sample size of the study was calculated using a single population for intention to use institutional delivery and a double population proportion for predictors. The final sample size of the study was 637 pregnant women. The multi-stage followed by cluster sampling technique was used to select study participants. Yilmana densa district has 11 clusters, of those 3 clusters were randomly selected using lottery method. All pregnant women in each cluster were included until the final sample size.

### Variables

#### Dependent variable

The dependent variable was intention to use institutional delivery service. It is the readiness/willingness of women to use institutional delivery service, it was measured with 3 items containing 5- point Likert scales. Those who scored mean and above categorized as intended to use institutional delivery service and whereas those who scored below mean categorized as not intended to use institutional delivery service [28].

#### Independent variables

Socio-demographic characteristics such as Age, marital status, educational status, monthly income, occupation, Husband’s occupation, Husband’s educational status and religion were included. The theory of planned behavior variables such as Attitude, subjective norm and perceived behavioral control, and Obstetric history factors like ANC visit, informed place of delivery, past experience of institutional delivery, and complications were included.

### Measurements

#### Attitude

The direct attitude was measured with 5 items using 5 point Likert scales ending with strongly disagree and strongly agree. Those who scored mean and above considered as having favorable attitude towards institutional delivery use. Whereas those who scored below mean considered as having unfavorable attitude towards institutional delivery service use [17]. The indirect attitude was measured by summing the products of behavioral beliefs with its corresponding outcome evaluation.

#### Subjective norm (SN)

An individual’s perception of using institutional delivery (ID), which is influenced by the judgment of significant others. It was measure with 5 items containing 5 point Likert scales ending with very bad and very good. Those who scored mean and above classified as favorable subjective norms towards institutional delivery use. Whereas those who scored below mean considered as unfavorable subjective norms towards institutional delivery use [16, 29]. Indirect subjective norm was measured by summing the product of normative belief with its corresponding motivation to comply.

#### Perceived behavioral control (PBC)

The individual’s belief concerning how easy or difficult of using institutional delivery (ID). It was measured with 5 items containing 5- point Likert scales those who scored mean and above classified as favorable perceived behavioral control towards institutional delivery use. Whereas those who scored below mean classified as unfavorable perceived behavioral control towards institutional delivery use [28]. The indirect perceived behavioral control was measured by summing product of control belief with its corresponding power.

### Data collection tools and procedures

Data was collected through a face-to-face interview with a structured and pretested questionnaire [15, 26–28]. The questionnaire were adapted from reviewing different literatures. Five data collectors with diploma holders with midwifery from health centers other than selected clusters and two supervisors with BSc in public health who spoke Amharic (local language) fluently were selected for data collection. To maintain data quality and consistencies the English version questionnaire was translated into Amharic (local language) for data collection by language experts. It was translated back to English. Pre-tested was conducted on 5% (32) of the final sample size in North Mecha district by principal investigator a week before actual data collection. A reliability test was performed for Likert scale questions by Cronbach’s alpha. The internal consistency by Cronbach’s alpha of direct attitude, direct subjective norm and direct perceived behavioral control was 0.84, 0.92 and 0.79 respectively.

### Data analysis

The data was entered using Epi-Data version 4.6 and analyzed using STATA version 14. Multivariable logistic regression analysis was performed to assess the association between socio-demographic character, obstetric, attitudes, subjective norm, perceived behavioral control, and experiences with intention to use institutional delivery service. Adjusted odds ratio was used as a measure of association 95% CI and p-value < 0.05 for statistical significant association of intention.

### Ethical clearance

Ethical clearance was obtained from institute of review board (IRB) of University of Gondar. From Yilmana Densa district health office, Letter of cooperation was written to each selected kebeles (the smallest administrative unit in Ethiopia) study participants. Following an explanation of the purpose of the study, consent was obtained from each participant. From each participant written consent was obtained and for those participants <18years old consent were obtained from their parents or guardian. Also, assure that they were free to withdraw consent and discontinue participation without any form of prejudices was made. Confidentiality of information and privacy of participants was assured for all the information provided.

## Results

### Sociodemographic characteristics

A total of 637 participants were included yielding 98.8% response rate. The mean age of the respondents with standard deviation was 28.7 years±5.78 years. Six hundred twenty-eight (98.6%) pregnant women were married. Three hundred fifty-three (55.4%) of the respondents had no formal education. Four hundred sixty-eight (73.5%) of women were living in rural areas. Three hundred eight (48.4%) of husbands had no formal education and 416(65.3%) of husbands were farmers (Table 1).

**Table 1:** Socio-demographic characteristics of pregnant women in Yilmana Densa district, West Gojam zone, northwest Ethiopia, 2020 (n = 637).

Variables	Categories	Frequency	Percent (%)
Age	15–19	11	1.7
	20–24	134	21
	25–29	233	36.6
	30–34	132	20.7
	>_35	127	19.9
Religion	Orthodox	607	95.3
	Muslim	30	4.7
Residence	Urban	169	26.5
	Rural	468	73.5
Marital status	Married	628	98.6
	Single	4	0.6
	Others	5	0.8
Educational status	No formal education	353	55.4
	Primary school	147	23.1
	Secondary school	91	14.3
	Diploma and above	46	7.2
Husband educational status	No formal education	308	48.4
	Primary school	184	28.9
	Secondary school	76	11.9
	Diploma and above	60	9.4
Women’s occupation	House wife	544	85.4
	Merchant	34	5.3
	Government employee	39	6.1
	Others	20	3.1
Husband occupation	Farmer	416	65.3
	Merchant	98	15.4
	Government employee	61	9.6
	Others	53	8.3
Monthly income	< = 500 ETB	87	13.7
	501–1000 ETB	296	46.5
	>1000 ETB	254	39.9

### Obstetric characteristics

Majority (87%) of the study participants had antenatal care and 82.7% reported that the current pregnancy was planned. About 327(51.3%) had 1–3 parity and 294(46.2%) respondents had past experience of institutional delivery. Eighty three (17.3%) participants were reported delivery-related complications before and (Table 2).

**Table 2:** Obstetric characteristics of pregnant women in Yilmana Densa district, northwest Ethiopia, 2020 (n = 637).

Variables	Categories	Frequency	Percent
Status of current pregnancy	Unplanned	110	17.3
	Planned	527	82.7
ANC visit	Yes	554	87
	No	83	13
Number of ANC visit	One	103	16.2
	Two	197	30.9
	Three	169	26.5
	Four and above	85	13.3
Gave birth before	Yes	474	74.4
	No	163	25.6
Parity	Zero	163	25.6
	1–3	327	51.3
	> = 4	147	23.1
Nearest past child condition	Alive	455	71.4
	Dead	19	3
Mode of former delivery	SVD	460	72.2
	CS	14	2.2
Informed about delivery places	Yes	362	56.8
	No	275	43.2
Types of delivery complication before	Excessive vaginal bleeding	29	48.3
	Retained placenta	13	20
	Eclampsia /preeclampsia	10	15.4
	Obstructed labor	31	47.7

### Magnitude of intention to use institutional delivery

About 74.3% (CI; 70.71%, 77.6%) of pregnant women intended to use institutional delivery service for their current pregnancy. The mean score of intention to use institutional delivery service was 11.58(SD±2.06).

### Theory of planned variables

The mean score of pregnant women’s direct and indirect attitude to use institutional delivery service was 19.46± 2.34 and 66.39±12.58 respectively. Majority 368(57.8%) the pregnant women had favorable direct attitude and less than one third 180(28.3%) of participants had favorable indirect attitude. The mean score of a direct and indirect subjective norm to use institutional delivery service among pregnant women was 19.04±2.82 and 60.56±16.18 respectively. The majority 413(64.8%) and more than half 353(55.4%) had favorable direct and indirect subjective norms respectively. The mean score of direct and indirect perceived behavioral control to use institutional delivery services was 18.33± 3.04 and 55.18±11.55. More than half 365(57.3%) and Three hundred thirty-three (53.1%) participants had favorable direct and indirect perceived behavioral control respectively (Table 3).

**Table 3:** Components of TPB among pregnant women in Yilmana Densa district, 2020.

Variables	Categories	Frequency	Percent	Mean±SD
DATT	Unfavorable	269	42.2	19.46± 2.34
	Favorable	368	57.8	
DSN	Unfavorable	224	35.2	19.04±2.82
	Favorable	413	64.8	
DPBC	Unfavorable	272	42.7	18.33± 3.04
	Favorable	365	57.3	
IATT	Unfavorable	457	71.7	66.39±12.58
	Favorable	180	28.3	
ISN	Unfavorable	284	44.6	60.56±16.18
	Favorable	353	55.4	
IPBC	Unfavorable	299	46.9	55.18±11.55
	Favorable	338	53.1	

### Multivariable logistic regression analysis

In the multivariable logistic regression; number of ANC visit, pas experience of institutional delivery, the residence of respondents, parity, attitude and perceived behavioral control were factors that showed statistically significant association with behavioral intention to use institutional delivery service after controlling for confounding factors.

Those who had 1–3 ANC visit were 2.85 times more intended to use institutional delivery service (AOR = 2.85,95%CI:1.41,5.75)and those who had 4 and above ANC visit for their current pregnancy were 3.14 times more intended to use institutional delivery service(AOR = 3.14, 95%CI:1.16, 8.45) as compared to those who had no visit.

The odds of intention to institutional delivery among pregnant women who had previous experience of institutional delivery were 3.4 times (AOR = 3.39, 95%CI: 1.72, 6.71) more likely to intended than those who had no past experience. Pregnant women who lived in rural residences were 49% times less likely intended to use institutional delivery services (AOR = 0.51, 95%CI: 0.27, 0.98) as compared to those who lived in urban residences.

The odds of intention to use institutional delivery service in those pregnant women who were with parity of 1–3 and 4 & above were 63% (AOR = 0.37, 0.19, 0.71) and 74% (AOR = 0.26, 95%CI: 0.12, 0.55) respectively less likely than those who had zero parity (who didn’t give birth before).

Those who had favorable attitude were 2.93 times more likely intended to use institutional delivery service (AOR = 2.93,95%CI:1.56,5.50) as compared to those who had an unfavorable attitude. Those who had favorable perceived behavioral control were 2.6 times more intended to use institutional delivery (AOR = 2.60, 98, 95%CI:1.44, 4.69) as compared to those who had unfavorable direct perceived behavioral control (Table 4).

**Table 4:** Multivariable logistic regression analysis of predictors of intention to use institutional delivery service among pregnant mothers in Yilmana Densa district, may, 2020 (n = 637).

Predictors	Intention		AOR(95%CI)	p-value
	Unintended	Intended		
Residence				
Urban	22	147	1	
Rural	142	326	0.51(0.27, 0.97)*	0.04
Women Educational status				
No formal education	131	222	1	
Primary school	18	129	1.45(.705,3.19)	0.29
Secondary and above	15	122	0.973(.365,2.56)	0.96
Husbands educational status				
No formal education	93	215	1	
Primary and above	67	253	0.773(.418,1.25)	0.41
M monthly income				
< = 500 ETB	27	60	1	
501–1000 ETB	86	210	1.28(.570, 2.88)	0.55
>1000 ETB	51	203	1.38(.560,3.41)	0.48
Pregnancy status				
Not planned	43	67	1	
Planned	121	406	0.61(.285,1.25)	0.21
Number of ANC visit				
No visit	56	27	1	
1–3 visit	96	373	2.85(1.41,5.75)*	0.004
4 and above visit	12	73	3.14(1.16, 8.45)*	0.02
Gave birth before				
No	32	131	1	
Yes	132	342	1.32(.05,33.36)	0.87
Parity				
0	32	131	1	
1–3	69	258	0.37(0.19, 0.71)*	0.003
>_4	63	84	0.26(0.12, 0.55)*	0.001
Informed place of delivery				
No	123	152	1	
Yes	41	321	1.14(0.62,2.08)	0.68
Previous ID use				
No	142	201	1	
Yes	22	272	3.39(1.72, 6.71)*	0.000
Attitude				
Unfavorable	139	130	1	
Favorable	25	343	2.93(1.56,5.50)*	0.001
Subjective norm				
Unfavorable	104	120	1	
Favorable	60	353	1.10(.59,2.04)	0.76
Perceived behavioral control				
Unfavorable	137	135	1	
Favorable	27	338	2.60(1.44, 4.69)*	0.001

*Statistical-significance at 95%CI and p<0.05.

## Discussion

The study aimed to assess intention to use institutional delivery service and its predictors among pregnant women in Yilmana Densa district. The result of this study showed that attitude, perceived behavioral control, number of ANC visit, past experience of institutional delivery, residence, and parity were factors showed significant association with intention to use institutional delivery service.

This study revealed that the magnitude of intention to use institutional delivery service was 473 (74.3%,95%CI:70.8,77.9). This study was in line with studies conducted in Debremarkos town [18], Dangila district [14], Debretabor town [30] and Wollaita Soddo town [6]. However, higher than the studies conducted in Afar pastoralist community [12] and in Yirgacheffie town [29]. This may be due to variations in the study area, period, and study population. This difference might also attributed to the multiple strategies nowadays implemented to increase institutional delivery.

This study showed that residence of the respondents was a statistically significant predictor of intention to use institutional delivery services. Those residing in rural dwelling were less likely to intend to use institutional delivery service as compared to urban residence. This finding is consistent with studies conducted in North Gondar [31] and Amhara region referral hospitals [32]. This result also in line with studies conducted in India [33] and Nepal [20]. The possible explanation may be women in rural area might be influenced by physical and informational barriers this might decrease intention to use institutional delivery service. Another explanation for this difference might be that more cultural influence in rural area like to use home delivery and embarrassment of pregnant women to show private parts to health professionals.

The current study revealed that the number of antenatal care (ANC) visit of the respondents was statistically significant predictor of intention to use institutional delivery service. The result was consistent with studies conducted in Nepal [20], Tanzania [25] and Pastoralist Communities of Afar [12]. The possible justification could be that pregnant Women who had frequent ANC visit may have a good opportunity to discuss with health professional about maternal complication and importance of institutional delivery as compared to those who had no visit. Another potential reason may be that ANC follows up by itself is one of maternal health service utilization which might influence the decision to use institutional delivery service. This was observed in other study antenatal care is a base for institutional delivery service [34].

In the present study, parity (number of delivery) of the respondents was found to be a statistically significant predictor of intention to use institutional delivery services. This might be mothers who had more number of children no more eager to have a child, give less attention to childbirth and they see the delivery of a child is easy. Another reason for this finding might be mothers with more children might have more home delivery habits [20].

This research revealed that past experience of institutional delivery service use was statistically significant predictor of intention to use institutional delivery services. Those who previously delivered in health facilities were more likely to use institutional delivery service for their current pregnancy compared to those who had no experience of institutional delivery use. It may be that those who used institutional delivery previously knew the advantages of health facilities delivery, had high awareness about maternal complications, and more confident to use institutional delivery services for their current pregnancy [23, 30].

The other statistically significant predictor of intention to use institutional delivery service was attitude. This study is in line with studies conducted in Wollaita Soddo town [13, 19] and Awash Fetale district [35]. This might be explained as those who had favorable attitude may have good knowledge of institutional delivery, which could increase their intention to use it [11, 36, 37].

Our finding showed that the subjective norm was not a statistically significant predictor of intention to use institutional delivery service. However, numerous studies for example, studies conducted in Wollaita Soddo town [6] and Zambia [23] showed that it had a statistically significant association with behavioral intention to use institutional delivery. This variation may be due to the empowerment of women, awareness creation towards institutional delivery, and women’s affirmative action become increased currently. This may reduce the influence of significant others such as husbands, families, and other groups on pregnant women to use institutional delivery service [38].

The current study revealed that perceived behavioral control was a statistically significant predictor of intention to use institutional delivery service. This study is in line with a studies conducted in Zambia [23] and Yirgacheffie town [29]. This may due to women’s understanding of delivery complications in-home delivery and women’s perception that they may not get supporters in their home during delivery. Another explanation might be that women believed that the availability of maternity waiting homes in health institutions is important to stay there before starting labor.

## Conclusion

Majority of the participants were intended to use institutional delivery for the current pregnancy. The statistically significant predictors of intention to use institutional delivery service were a place of residence, parity, number of ANC, past experience, attitude, and perceived behavioral control. Therefore, more attention should be given for rural women and information education communication & behavior change communication activities are needed to increase number of ANC visit users, change attitude and to overcome barriers so that they could have more intention to institutional delivery utilization.

## Supporting information

S1 Data (DTA)

## Abbreviations and acronyms

ANC: Antenatal care
WHO: World health organization
AOR: Adjusted odds ratio
TPB: Theory of planned behavior
APH: Antepartum hemorrhage
SD: Standard deviation
ATT: Attitude
SSA: Sub Saharan Africa
CI: Confidence interval
PPH: postpartum hemorrhage
CS: Caesarian section
PBC: Perceived behavioral control
ETB: Ethiopian birr
ID: Institutional delivery
